# The Role of Social Support for Caregivers of Persons Living with Dementia (PLwD) in Hospice: A Secondary Analysis

**DOI:** 10.1002/alz.088439

**Published:** 2025-01-09

**Authors:** Hannah Cho, Liming Huang, Debra Parker Oliver, Karla Washington, George Demiris

**Affiliations:** ^1^ University of Pennsylvania, Philadelphia, PA USA; ^2^ Washington University in St. Louis, St. Louis, MO USA

## Abstract

**Background:**

There are 55 million persons living with dementia (PLwD) today, projected to 139 million by 2050. As they progress towards dementia’s advanced stages, hospice care becomes crucial to manage their symptoms and the caregiver burden. Solo family caregivers, particularly lacking in social support, are vulnerable to deteriorating physical and mental well‐being. Previous studies highlighted adequate social support as one factor consistently related to better caregiver outcomes; yet there is a significant gap in our understanding of their experiences in hospice settings. This study examines the moderating factors for hospice family caregivers, exploring the relationship between social support, anxiety, depressive symptoms, and physical health.

**Method:**

This is a secondary analysis of baseline data from an ongoing study testing a behavioral intervention for caregivers. Participants were adult caregivers of PLwD who completed the Generalized Anxiety Disorder – 7, the Patient Health Questionnaire – 9, the Medical Outcome Study‐ Social Support Survey, and demographic information. A statistical analysis including descriptive statistics, Pearson correlation and linear regressions was performed.

**Result:**

One hundred fifty‐seven adults were enrolled in the time frame of this study. Most caregivers were female (63.05%) and average age was 60 (SD 13.52). By regressing physical health, anxiety and depressive symptoms on social support respectively, we found that having more social support is significantly associated with less depressive symptoms (b = ‐.047, *p = *.029) and improved physical health (b = .273, *p* = .0001) and the direction and significance of these relationships still hold after adjusting for employment changes (i.e., quitting or reducing working hours) (*p* = .013, *p* = 0.001). Controlling for caregiver age, depressive symptoms decrease when social support increases (b = ‐.053, *p* = .034).

**Conclusion:**

In a hospice setting, inadequate social support significantly elevates depression risk for caregivers of PLwD. Being a primary caregiver, having more hours of caregiving, and seeking support and resources from reducing or quitting a job could affect their physical and mental well‐being. Further examination of these findings will inform supportive strategies for families of PLwD.

